# TEACHING SEX AND AGING TO PRE-PROFESSIONALS: EXPERIENTIAL LEARNING OF A TABOO TOPIC

**DOI:** 10.1093/geroni/igz038.1977

**Published:** 2019-11-08

**Authors:** Jill Juris Naar, Raven H Weaver, Libbie Sonnier-Netto

**Affiliations:** 1 Appalachian State University, Boone, North Carolina, United States; 2 Washington State University, Pullman, Washington, United States; 3 Center for Development and Learning/Virginia Tech, New Orleans, Louisiana, United States

## Abstract

Myths and stereotypes of sex in late life prevail (e.g., sex becomes unimportant or irrelevant; sex gets worse as individuals age; older adults are asexual). Unless the workforce is adequately prepared and knowledgeable to address the sexual health needs of older adults, it is difficult to disrupt ageism that contribute to discrimination against older adults. Methods: We evaluated a two-day experiential workshop designed for students to develop factual community resources and educational materials related to sex and aging. Results: Using content analysis, we examined open-ended responses from 41 students. We identified three themes that depicted the value of experiential learning, specifically for this taboo topic. Students (1) increased awareness of late life sexual health and behaviors, (2) demonstrated comfort and creative strategies to discuss sex, and (3) appreciated the opportunity for transferable pre-professional skill development. Discussion: Providing pre-professional training helped close the knowledge gap about basic gerontological issues and issues specific to sexual health and sexual behaviors in late life. Students perceived the flipped classroom and collaborative structure of this experiential workshop as beneficial and practical for their learning and professional preparation. They developed skills for translating knowledge into practical resources that likely will transcend professions. Emphasizing the potentially uncomfortable topic of sex and aging provided students an opportunity to increase their comfort when addressing issues they will experience in their various professions. It is critical for gerontology educators to identify strategies to deliver sex and aging education among professionals to enhance interactions with older adults.

